# Response to: Comment #2 on “Differences in Ventilatory Threshold for Exercise Prescription in Outpatient Diabetic and Sarcopenic Obese Subjects”

**DOI:** 10.1155/2018/3093208

**Published:** 2018-12-24

**Authors:** Gian Pietro Emerenziani, Maria Chiara Gallotta, Silvia Migliaccio, Emanuela A. Greco, Chiara Marocco, Luca di Lazzaro, Rachele Fornari, Andrea Lenzi, Carlo Baldari, Laura Guidetti

**Affiliations:** ^1^Department of Experimental and Clinical Medicine, University of Magna Graecia of Catanzaro, Catanzaro, Italy; ^2^Department of Movement, Human and Health Sciences, University of Rome “Foro Italico”, Rome, Italy; ^3^Department of Experimental Medicine, Sapienza University of Rome, Rome, Italy; ^4^LiSa Laboratory, Policlinico of Catania, University of Catania, Catania, Italy; ^5^University eCampus, Novedrate, Italy

We would like to thank Goran Kuvačić and Jhonny Padulo for being still interested in our study and Luca Paolo Ardigò for his comments [1]. We regret that Dr. Kuvačić and Dr. Padulo believed that our previous response to their comments [2] did not fully satisfy their concerns [3]. We hope that this new response will fully address authors' doubts. We include our answers below.

*Point 1*. We would like to remind readers that the main aim of our study [4] was to evaluate the subjects' individual ventilatory threshold in obese older adults. As indicated by Simonton et al. [5], a rapid exercise protocol (increment every 1 min) was superior to the gradual protocol (increment every 3 min) for determination of ventilatory threshold in normal subjects. In contrast, the reproducibility of the gradual protocol was slightly higher in the patient, producing not significantly different results from the rapid protocol. Since our main aim was to evaluate the ventilatory threshold in patients, we choose a gradual protocol. We take note that authors disagree with the use of a modified Balke protocol to assess peak oxygen uptake ($\dot{V}$O_2peak_) in obese subjects, but neither “Balke protocol” nor “modified Balke protocol” was mentioned in the article [4]. In the article [4], the maximal effort exercise protocol was described as follows: “treadmill protocol started at 3 km/h and then speed increased by 1 km/h every two minutes until 5 km/h was reached. Then, slope was increased by 3% every two minutes until subjects reached a value of 10 on RPE scale.” This is a synthetic description that allows reproduction of the protocol. Since the criterion to stop exercise testing was only the perceived maximum RPE, the $\dot{V}$O_2_ assessed was termed “$\dot{V}$O_2peak_” as suggested by McArdle et al. [6].

We would like to note that in [1], the authors affirm that the use of the Balke protocol (or any modified Balke protocol) should be avoided in order to achieve the real $\dot{V}$O_2peak_, as indicated in reference number 6 of [7] and reference number 7 of [8], in support of this statement. The subjects studied in the article published by da Silva et al. [7] were healthy young adults (age 29.1 ± 7.6 yrs), and no one subject was obese. Therefore, the conclusions of the study [7] might not be applicable to the obese population. The aim of the study [8] was to analyse whether obese adults meet the criteria to assess $\dot{V}$O_2max_ during an incremental exercise test, and there are no data or comments regarding the subjects' $\dot{V}$O_2peak_ or IVT. Moreover, other scientific articles used a modified Balke protocol to assess $\dot{V}$O_2peak_ in obese subjects [9, 10].

Based upon these observations, we believe that the point raised by Kuvačić et al. [1] is not based upon specifically related scientific evidence.

*Point 2*. In [4], data regarding the test-retest precision of the measure were not reported. The precision of the measure was as follows: coefficient of variation (CV) is lower than 5% and the intraclass correlation coefficient (ICC) is higher than 0.97 (indicating a very good reliability of the treadmill protocol). Data regarding the CVs and ICCs of our continuous incremental treadmill test protocol are also shown in the recent published study [11].

*Point 3*. The treadmill model Woodway Pro (Woodway, Waukesha, WI, USA) was used. The maintenance and calibration of the treadmill were done according to the user manual [12] and carried out by trained and authorized personnel.

In summary, we further believe that the methods used in our previous manuscript [4] are scientifically correct and appropriate for fitness evaluation in obese subjects. As mentioned above, the procedure of treadmill exercise test used to evaluate subjects' obese fitness is also described in further detail in a recent manuscript [11].
